# Editorial: Altered epigenetic modification mediated dysregulated transcriptional networks in drug resistance

**DOI:** 10.3389/fonc.2023.1196448

**Published:** 2023-04-21

**Authors:** Bryce Brunetti, Akihiro Yoshida

**Affiliations:** ^1^ Department of Dermatology, Case Western Reserve University and University Hospitals Cleveland Medical Center, Cleveland, OH, United States; ^2^ Case Comprehensive Cancer Center, Cleveland, OH, United States

**Keywords:** cancer, dysregulated transcription, drug resistance, epigenetic modification, epigenetic, transcriptional network

Many types of chemotherapy have been developed and successfully used to treat cancer patients. However, some chemotherapy targeting cancers with a specific mutation may become ineffective as cancer cells develop new strategies of drug resistance. Epigenetic modifications that cause amplification of oncogenes, and inactivation of tumor suppressors, have emerged as key factors in drug resistance. This editorial summarizes several studies that have identified novel dysregulated transcriptional networks that can be targeted such as histamine-induced cell proliferation, gene silencing, and transcriptional dysregulation by circRNA. Each paper shows future potential for better outcomes for patients with drug resistant tumors.

Ovarian cancer (OC) ranks first in mortality for gynecological tumors with high recurrence and minimal treatment options. Although the complete pathogenesis of OC remains unclear, it is evident that transcriptional modifications, such as the upregulation of estrogen receptors (ERs) by histamine, promote the proliferation of OC cells (Liu et al.). The recent study by Liu et al. investigated the potential function of apigenin in treating OC. Apigenin, a natural flavonoid, shown to inhibit the proliferation of histamine from basophils and inhibit cell proliferation in many tumor types (1). Mast cells (MC), are known to play a key role in immune-inflammatory response via the secretion of bioactive molecules, primarily histamine, which has been linked to promoting tumor growth (2). Liu et al. examined histamine levels in OC tissues with that of normal ovarian tissue by immunohistochemical staining and identified statistically significant (*P<*0.05) higher levels of histamine in OC tissues compared to normal. They found that OC cells treated with 50 ng/mL histamine for 48 hours had statistically significant (*P<*0.05) higher levels of proliferation compared to untreated OC cells. These findings suggest that MC increased release of histamine, could expedite OC progression. Findings also showed that histamine promotes OC cell proliferation by regulating ER expression. ERs play a vital part in estrogen-dependent tumors regulating signaling pathways known to aid in angiogenesis, cell invasion, migration, differentiation, and proliferation, specifically PI3K, p-AKT, and p-mTOR. Liu et al. further studied ERα and ERβ thought to be tumor-inductive and tumor-suppressive respectively, and found elevated levels of proliferation in the incidence of ERα and inhibited by ERβ. Given that apigenin has been found to have estrogen-like properties, they tested the efficacy of apigenin and demonstrated that although apigenin did not directly affect the expression of histamine receptors H1 and H3, instead reversed the overexpression of ERα and inhibition of ERβ on both transcriptional and translational levels in tumor cells. Regulation of ER expression by apigenin also suppressed signaling pathways overexpression of PI3K, p-AKT, and p-mTOR, resulting in increased tumor cell apoptosis and decreased cancer proliferation. In summary, apigenin appears to suppress histamine overexpression of the ER, decreased OC tumor development, and shows promise for use in inhibiting OC progression (**Figure 1**).

**Figure 1:**
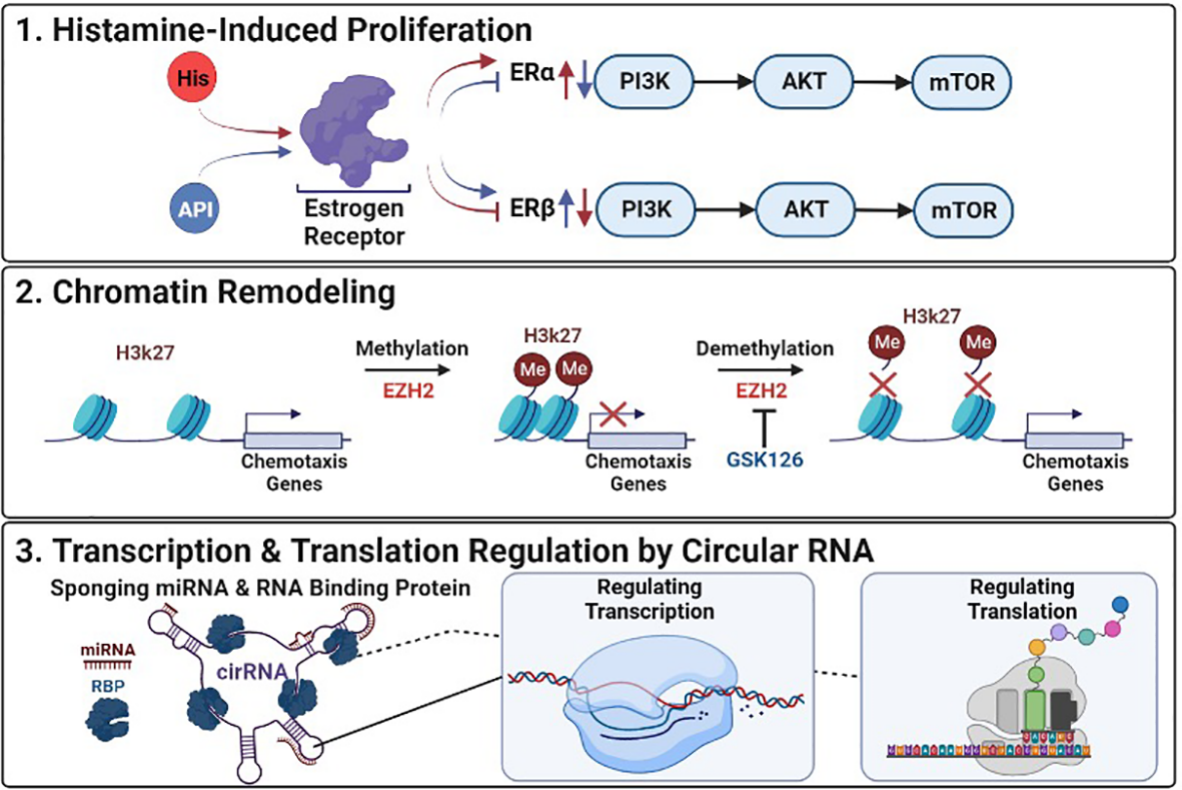
Epigenetic Deregulation in Cancer: (1) Histamine-Induced Proliferation: Histamine (HIS) upregulates ERα and downregulates ERβ promoting proliferation and tumor development, Apigenin (API) inhibits ERα and upregulates ERβ suppressing histamines effects. (2) Chromatin Remodeling: GSK126 inhibits EZH2 reversing transcriptional repression of genes by preventing the methylation of histone at H3K27. (3) Transcription & Translation Regulation by Circular RNA: Mechanisms by which circular RNAs (cirRNA) can alter transcription and translation of targeted genes through interactions with mircoRNAs (miRNA) and RNA binding proteins (RBP).

Epigenetic modifications such as histone methylation have been shown to be one of the key methods used by glioblastoma (GBM) tumor cells to silence gene expression and evade immune cells (3). The study by Ratnam et al. investigated treating GBM, previously thought to have brain-immune privileges, with immunotherapy. However, due to histone methylation silencing genes that promote chemotaxis, which aid in trafficking lymphocytes like T cells to the tumor, the efficacy of immunotherapy in treating glioblastoma has been limited (4). To improve the efficacy of immunotherapy Ratnam et al. examined GSK126, a small molecule able to pass the blood-brain barrier (BBB), and reverse histone methylation disruption of T cell chemotaxis via inhibition of EZH2, a histone methyltransferase (5), and suppress solid tumors in GBM. They found that patients with GBM tumors had limited T cell infiltration and that T cells isolated from tumors had elevated expression of CXCR3, a chemokine receptor that depends on CXCL9/10 for trafficking T cells from the periphery to the brain, compared to T cells from healthy donors. Furthermore, they treated GBM cell lines with GSK126 or a combination with IFNγ, an inducer of CXCL9/10, and demonstrated that some cell lines treated with GSK126 had a greater reversal of histone methylation, measured by H3K27me3 expression, and combination treatment increased migration of T cells in tumor conditioned medium. They further demonstrated that immunocompetent mice treated with GSK126 decreased H3K27me3 expression, and suppressed intracranial tumor growth. In addition, they investigated the mode of action of combinatorial treatment of GSK126 with an immune checkpoint inhibitor an anti-PD-1 antibody, in a subcutaneous tumor model absent of BBB and observed decreased H3K27me3 with no significant change in EZH2 expression between groups. These findings are consistent with the proposed mechanism of GSK126’s ability to inhibit EZH2 methylation, reversing chemotaxis gene silencing, and suggest that GSK126s can lead to more effective treatments for patients with GMB (**Figure 1**).

This topic also includes two review articles examining the emerging impact of circular RNAs (circRNA) in the context of drug resistance. As research into drug resistance in cancer progresses more studies point to circRNA as a significant factor. The review of Wang et al. identifies several reported mechanisms of circRNA which may directly or indirectly lead to drug resistance. One mechanism describes circRNA binding microRNAs (miRNA), a non-coding RNA that aids in regulating gene expression, competing with the intended target gene effectively suppressing the effects of miRNA in regulating gene expression. In contrast, circRNAs have also been found to efficiently upregulate mRNA and protein levels leading to chemoresistance in some instances. The review by Ghazimoradi and Babashah, relayed that circRNA “sponging” of miRNA, and microRNA was observed to be one of the most abundant and deregulated mechanisms in cancer. They review multiple studies where circRNA-suppressing miRNA interferes with tumor suppressors and the silencing of oncogenes aiding in drug resistance. In addition to miRNA sponging, both reviews discussed findings that circRNA can bind RNA-binding proteins further disrupting cellular processes and downstream signaling pathways. In addition, both reviews collaborated that the tumor type and microenvironment greatly affected the function of the same circRNA. Even though more research is needed into the impactful pathways circRNA disrupt, both studies suggest that CircRNA/miRNA/mRNA could be used as potential treatment targets (**Figure 1**).

In summary, these studies identify novel mechanisms and pathways with the potential to reverse or target epigenetic modifications, and aid in the treatment of multidrug resistance as summarized in **Figure 1**. As researchers continue to study these transcriptional networks connected to drug resistance, more discoveries may lead to better targets for cancer treatment, and outcomes for cancer patients.

## Author contributions

BB and AY conceived and wrote the manuscript. All authors contributed to the article and approved the submitted version.

## References

[1] Mafuvadze B. Liang Y. Besch-Williford C.. Apigenin Induces Apoptosis and Blocks Growth ofMedroxyprogesterone Acetate-Dependent BT-474 Xenograft Tumors. HORM CANC (2012) 3:160–71. doi: 10.1007/s12672-012-0114-x PMC10358033 22569706

[2] Kennedy L Hodges K Meng F Alpini G Francis H. Histamine and histamine receptor regulation of gastrointestinal cancers. Transl Gastrointest Cancer (2012) 1(3):215–27. doi: 10.1007/s12672-012-0114-x PMC3955103 24639917

[3] Gabrusiewicz K Rodriguez B Wei J Hashimoto Y Healy LM Maiti SN. Glioblastoma-infiltrated innate immune cells resemble M0 macrophage phenotype. JCI Insight (2016) 1(2). doi: 10.1172/jci.insight.85841 PMC4784261 26973881

[4] Carson MJ Doose JM Melchior B Schmid CD Ploix CC. CNS immune privilege: hiding in plain sight. Immunol Rev (2006) 213:48. doi: 10.1111/j.1600-065X.2006.00441.x 16972896 PMC2633103

[5] Zingg D Arenas-Ramirez N Sahin D Rosalia RA Antunes AT Haeusel J. The histone methyltransferase Ezh2 controls mechanisms of adaptive resistance to tumor immunotherapy. Cell Rep (2017) 20(4):854–67. doi: 10.1016/j.celrep.2017.07.007 28746871

